# Machine learning score to predict in-hospital outcomes in patients hospitalized in cardiac intensive care unit

**DOI:** 10.1093/ehjdh/ztae098

**Published:** 2024-12-20

**Authors:** Orianne Weizman, Kenza Hamzi, Patrick Henry, Guillaume Schurtz, Marie Hauguel-Moreau, Antonin Trimaille, Marc Bedossa, Jean Claude Dib, Sabir Attou, Tanissia Boukertouta, Franck Boccara, Thibaut Pommier, Pascal Lim, Thomas Bochaton, Damien Millischer, Benoit Merat, Fabien Picard, Nissim Grinberg, David Sulman, Bastien Pasdeloup, Yassine El Ouahidi, Treçy Gonçalves, Eric Vicaut, Jean-Guillaume Dillinger, Solenn Toupin, Théo Pezel, ADDICT-ICCU Investigators, Victor Aboyans, Emeric Albert, Franck Albert, Sean Alvain, Nabil Amri, Stéphane Andrieu, Sabir Attou, Simon Auvray, Sonia Azzakani, Ruben Azencot, Marc Bedossa, Franck Boccara, Albert Boccara, Thomas Bochaton, Eric Bonnefoy-Cudraz, Guillaume Bonnet, Guillaume Bonnet, Nabil Bouali, Océane Bouchot, Claire Bouleti, Tanissia Boukertouta, Jean Baptiste Brette, Marjorie Canu, Aures Chaib, Clement Charbonnel, Anne Solene Chaussade, Alexandre Coppens, Yves Cottin, Arthur Darmon, Elena De ANGELIS, Clément Delmas, Laura Delsarte, Antoine Deney, Jean Claude Dib, Jean-Guillaume Dillinger, Clemence Docq, Valentin Dupasquier, Meyer Elbaz, Antony El Hadad, Amine El Ouahidi, Nacim Ezzouhairi, Julien Fabre, Damien Fard, Charles Fauvel, Édouard Gerbaud, Martine Gilard, Marc Goralski, Nissim Grinberg, Alain Grentzinger, Marie Hauguel-Moreau, Patrick Henry, Fabien Huet, Thomas Landemaine, Benoit Lattuca, Léo Lemarchand, Thomas Levasseur, Pascal Lim, Laura Maitre Ballesteros, Nicolas Mansencal, Benjamin Marie, David Martinez, Benoit Merat, Christophe Meune, Damien Millischer, Thomas Moine, Pascal Nhan, Nathalie Noirclerc, Patrick Ohlmann, Théo Pezel, Fabien Picard, Nicolas Piliero, Thibaut Pommier, Etienne Puymirat, Arthur Ramonatxo, Reza Rossanaly Vasram, François Roubille, Vincent Roule, Guillaume Schurtz, Mathilde Stevenard, David Sulman, Fédérico Swedsky, Victoria Tea, Eugénie Thevenet, Christophe Thuaire, Antonin Trimaille, Christophe Tron, Guillaume Viboud, Dominique Yomi, Cyril Zakine

**Affiliations:** Department of Cardiology, APHP-Hopital Ambroise Paré, 92100 Boulogne Billancourt, France; Université Paris-Cité, PARCC, INSERM, 75015 Paris, France; Department of Cardiology, University Hospital of Lariboisiere (Assistance Publique des Hôpitaux de Paris, AP-HP), Université Paris-Cité, Inserm MASCOT UMRS 942, 2 Rue Ambroise Paré, 75010 Paris, France; DATA-TEMPLE Laboratory, Department of Data Science, Machine Learning and Artificial Intelligence in Health, University Hospital of Lariboisiere (AP-HP), 2 Rue Ambroise Paré, 75010 Paris, France; Department of Cardiology, University Hospital of Lariboisiere (Assistance Publique des Hôpitaux de Paris, AP-HP), Université Paris-Cité, Inserm MASCOT UMRS 942, 2 Rue Ambroise Paré, 75010 Paris, France; DATA-TEMPLE Laboratory, Department of Data Science, Machine Learning and Artificial Intelligence in Health, University Hospital of Lariboisiere (AP-HP), 2 Rue Ambroise Paré, 75010 Paris, France; Department of Cardiology, University Hospital of Lille, Lille, France; Department of Cardiology, APHP-Hopital Ambroise Paré, 92100 Boulogne Billancourt, France; Department of Cardiology, Nouvel Hôpital Civil, Strasbourg University Hospital, 67000 Strasbourg, France; Department of Cardiology, CHU Rennes, 35000 Rennes, France; Department of Cardiology, Clinique Ambroise Paré, Neuilly-sur-Seine, France; Department of Cardiology, Caen University Hospital, Caen, France; Department of Cardiology, Hôpital Avicenne, Assistance Publique-Hôpitaux de Paris, Paris, France; Department of Cardiology, Saint-Antoine Hospital, APHP, Sorbonne University, Paris, France; Department of Cardiology, University Hospital, Dijon, France; Intensive Cardiac Care Department, University Hospital Henri Mondor, 94000 Créteil, France; Intensive Cardiological Care Division, Louis Pradel Hospital, Hospices Civils de Lyon, Bron, France; Cardiology Department, Montfermeil Hospital, 93370 Montfermeil, France; Cardiology and Aeronautical Medicine Department, Hôpital d'Instruction des Armées Percy, 101 Avenue Henri Barbusse, 92140 Clamart, France; Cardiology Department, Hôpital Cochin, Paris, France; Cardiology Department, Hôpital Mignot, Versailles, France; Department of Cardiology, Hôpital Bichat, Assistance Publique-Hôpitaux de Paris, Université de Paris, Paris, France; IMT Atlantique, Lab-STICC, UMR CNRS 6285, 29238 Brest, France; IMT Atlantique, Lab-STICC, UMR CNRS 6285, 29238 Brest, France; Department of Cardiology, University Hospital of Lariboisiere (Assistance Publique des Hôpitaux de Paris, AP-HP), Université Paris-Cité, Inserm MASCOT UMRS 942, 2 Rue Ambroise Paré, 75010 Paris, France; DATA-TEMPLE Laboratory, Department of Data Science, Machine Learning and Artificial Intelligence in Health, University Hospital of Lariboisiere (AP-HP), 2 Rue Ambroise Paré, 75010 Paris, France; Unité de Recherche Clinique, Groupe Hospitalier Lariboisiere Fernand-Widal, Paris, Île-de-France, France; Department of Cardiology, University Hospital of Lariboisiere (Assistance Publique des Hôpitaux de Paris, AP-HP), Université Paris-Cité, Inserm MASCOT UMRS 942, 2 Rue Ambroise Paré, 75010 Paris, France; DATA-TEMPLE Laboratory, Department of Data Science, Machine Learning and Artificial Intelligence in Health, University Hospital of Lariboisiere (AP-HP), 2 Rue Ambroise Paré, 75010 Paris, France; Department of Cardiology, University Hospital of Lariboisiere (Assistance Publique des Hôpitaux de Paris, AP-HP), Université Paris-Cité, Inserm MASCOT UMRS 942, 2 Rue Ambroise Paré, 75010 Paris, France; DATA-TEMPLE Laboratory, Department of Data Science, Machine Learning and Artificial Intelligence in Health, University Hospital of Lariboisiere (AP-HP), 2 Rue Ambroise Paré, 75010 Paris, France; Department of Cardiology, University Hospital of Lariboisiere (Assistance Publique des Hôpitaux de Paris, AP-HP), Université Paris-Cité, Inserm MASCOT UMRS 942, 2 Rue Ambroise Paré, 75010 Paris, France; DATA-TEMPLE Laboratory, Department of Data Science, Machine Learning and Artificial Intelligence in Health, University Hospital of Lariboisiere (AP-HP), 2 Rue Ambroise Paré, 75010 Paris, France

**Keywords:** Machine learning, Intensive cardiac care unit, Echocardiography, Outcomes, Death

## Abstract

**Aims:**

Although some scores based on traditional statistical methods are available for risk stratification in patients hospitalized in cardiac intensive care units (CICUs), the interest of machine learning (ML) methods for risk stratification in this field is not well established. We aimed to build an ML model to predict in-hospital major adverse events (MAE) in patients hospitalized in CICU.

**Methods and results:**

In April 2021, a French national prospective multicentre study involving 39 centres included all consecutive patients admitted to CICU. The primary outcome was in-hospital MAE, including death, resuscitated cardiac arrest, or cardiogenic shock. Using 31 randomly assigned centres as an index cohort (divided into training and testing sets), several ML models were evaluated to predict in-hospital MAE. The eight remaining centres were used as an external validation cohort. Among 1499 consecutive patients included (aged 64 ± 15 years, 70% male), 67 had in-hospital MAE (4.3%). Out of 28 clinical, biological, ECG, and echocardiographic variables, seven were selected to predict MAE in the training set (*n* = 844). Boosted cost-sensitive C5.0 technique showed the best performance compared with other ML methods [receiver operating characteristic area under the curve (AUROC) = 0.90, precision–recall AUC = 0.57, *F*1 score = 0.5]. Our ML score showed a better performance than existing scores (AUROC: ML score = 0.90 vs. Thrombolysis In Myocardial Infarction (TIMI) score: 0.56, Global Registry of Acute Coronary Events (GRACE) score: 0.52, Acute Heart Failure (ACUTE-HF) score: 0.65; all *P* < 0.05). Machine learning score also showed excellent performance in the external cohort (AUROC = 0.88).

**Conclusion:**

This new ML score is the first to demonstrate improved performance in predicting in-hospital outcomes over existing scores in patients admitted to the intensive care unit based on seven simple and rapid clinical and echocardiographic variables.

**Trial Registration:**

ClinicalTrials.gov Identifier: NCT05063097.

## Graphical Abstract

**Graphical Abstract:**
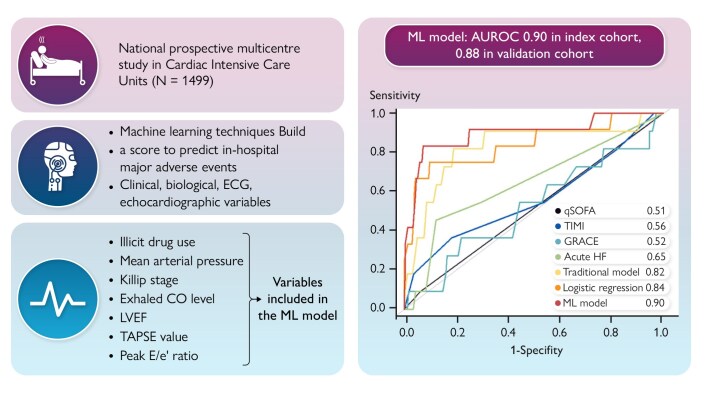

## Introduction

Machine learning (ML) is a subdomain of artificial intelligence, where a model is trained to produce predictions, using a large quantity of generally multivariable data.^1^ The large amount of information to be processed in the medical setting makes it an optimal domain for the use of artificial intelligence methods. ML techniques handle the complexity of the available data optimizing predictive performance and decision-making in several diseases.^2,3^ In the field of cardiovascular health in recent years, ML has been shown to be of particular interest.^4,5^ Cardiovascular imaging is particularly suited to these techniques because of the large amount of data to be processed, as recently studied by our working group.^6^

The mortality rate for patients hospitalized in cardiac intensive care units (CICUs) has been estimated at 5–8%, which is not negligible. For the clinician, risk stratification and prediction are important to adapt clinical and biological monitoring. The ability of ML to integrate multimodal clinical, biological, electrocardiographic, and echocardiographic data seems of particular interest in the critical care setting.^7,8^ However, risk stratification using ML techniques in CICU has been poorly evaluated.^9^

Therefore, this study aimed to assess the feasibility and performance of a ML-based score, through a large multicentre registry of consecutive patients admitted to CICU, to predict in-hospital major adverse event (MAE). The performance of this ML score will then be compared with existing scores and evaluated in an external validation cohort.

## Methods

### Study population

The ADDICT-CICU study is a prospective, multicentre cohort study evaluating the prevalence of psychoactive drug use and its prognostic impact in patients hospitalized for acute cardiac events. The study design of the ADDICT-CICU has been described in details previously.^10^ In summary, all consecutive adults admitted to CICUs for 2 weeks in April 2021 among 39 centres across France were included. Cardiac intensive care units are units that allow hospitalization of all patients with acute cardiovascular disease including acute coronary syndrome (ACS), acute heart failure (HF), and other cardiovascular emergencies. The list of participating centres is provided in Supplementary material online, *Methods S1*. The inclusion criteria were as follows: male or female patients aged ≥18 years admitted to the CICU whatever the medical reason with a written informed consent obtained at enrolment. The management of each patient was at the discretion of the treating physicians following the current European Society of Cardiology guidelines.^11–13^ This study was approved by the Ethics Committee (Committee for the Protection of Human Subjects, Ile de France-7, France) and registered on the Clinicaltrials.gov website under the number NCT05063097.

### Baseline characteristics collection

All details regarding baseline characteristics collection were already published.^10^ Briefly, baseline data included clinical, reason for hospitalization, list of medications, especially cardiovascular drugs at admission, and history of cardiovascular disease. Haemodynamic was evaluated using non-invasive measurement of arterial pressure. Transthoracic echocardiography was performed systematically within the first 24 h of admission for all patients (all standardized echocardiographic parameters are presented in the Supplementary material online, *Methods S3*). Biological data were collected systematically upon admission, including haemoglobin, serum potassium, creatinaemia, the peak of troponin, the N-terminal prohormone of B-type natriuretic peptide, or B-type natriuretic peptide.

The presence of illicit drugs was determined through urine analysis using a dedicated drug assay (NarcoCheck®, Kappa City Biotech SAS, Montluçon, France) within 2 h of admission to the CICU. The following illicit drugs will be screened for all consecutive patients: (i) cannabinoids (tetrahydrocannabinol), including cannabis and hashish; (ii) cocaine and metabolites, including cocaine and crack; (iii) amphetamines; (iv) 3,4-methylenedioxy-methylamphetamine (or ecstasy); and (v) heroin and other opioids. The NarcoCheck® urine drug assay detected the following medical psychoactive drugs: barbiturates, benzodiazepines, tricyclic antidepressant drugs, methadone, and buprenorphine (see Supplementary material online, *Methods S2*). A standardized exhaled carbon monoxide (CO) measurement was systematically taken with a CO-Check Pro device (Micro Direct Diagnostics Ltd, UK) immediately on arrival in CICU. The threshold of >10 parts per million was used to signify an active smoking.^14^

### Primary outcome

The primary outcome was in-hospital MAE defined as a composite of all-cause in-hospital mortality, cardiogenic shock (requiring medical or mechanical haemodynamic support), and resuscitated cardiac arrest (severe ventricular arrhythmia requiring defibrillation or anti-arrhythmic agents). All events, included in-hospital MAE, were adjudicated by an independent committee of experts who reviewed anonymized medical documents according to standardized definitions.^15^

### Cohort split

The ADDICT-CICU trial contains specific variables such as CO level and illicit drug use using a systematic urinary assay that are difficult to find in other databases. To build an independent validation cohort and assess the reproducibility of the ML model, we selected centres from the ADDICTO-USIC trial. Centres were randomly selected so that the total population of this cohort represented 20% of the ADDICTO-USIC trial population (8 centres, *n* = 294 patients), the remaining 80% constituting the index cohort (see Supplementary material online, *Table S1*, 31 centres, *n* = 1205 patients). Then, several supervised ML models were trained on 70% of the patients in the index cohort (*n* = 844) using a single uniform random split at a patient level and subsequently evaluated on the remaining 30% for internal validation across all models (*n* = 361 patients).

### Feature selection

Feature selection is the first step in ML analysis and was conducted according to the PRIME check list.^16^ A dedicated algorithm pre-evaluates the importance of the variables in explaining the desired phenomenon. Variables whose role in predicting the event exceeds 5% are selected for further development of the ML model. After excluding variables by collinearity criteria, 28 variables were considered for the Boruta variable selection algorithm (see Supplementary material online, *Table S2*): age, body mass index, sex, oxygen saturation, dyslipidaemia, left ventricle dilatation, psychiatric history, left atrium dilatation, heart rate, hypertension, family history of coronary artery disease, diabetes, alcohol consumption, active cancer, left ventricle dysfunction, right ventricle dilatation, N-terminal prohormone of brain natriuretic peptide, known coronary artery disease, creatinaemia, troponin peak, haemoglobin, drug consumption, ratio *E*/*e*′, Killip class, left ventricular ejection fraction (LVEF), mean arterial pressure, CO level, and tricuspid annular plane systolic excursion (TAPSE). The Boruta process consists of adding randomness to a given data set by creating mixed copies of all features, called shadow features.^17^ A random forest classifier applies feature importance measure to the augmented data set. At each iteration, it tests whether the true feature is more important than its shadow feature, considering a *P*-value < 0.05, and constantly removes unimportant features. Missing data in variables selected by the Boruta technique are given in Supplementary material online, *Table S3*.

### Data preprocessing

Various data preprocessing approaches were investigated within the scope of this study. The data set underwent manipulation through diverse methodologies aimed at assessing and enhancing algorithmic performance. Initially, the primary data set was adjusted to exclude patients with missing data, and no preprocessing steps were applied. The primary findings were based on this approach. In the second approach, we imputed missing values using the multivariate imputation by chained equations method (R package: mice). Finally, to address imbalanced date (low prevalence of events), we used a third strategy of data resampling with the synthetic minority oversampling technique algorithm (R package: DMwR).

### Model selection and evaluation

In the context of imbalanced data (a large number of patients for a small number of events), a comparative analysis was conducted among logistic regression and alternative supervised ML algorithms (including ridge regression, boosted cost-sensitive C5.0, and XGBoost). While logistic regression faces challenges with imbalanced data, ridge regression introduces regularization to mitigate overfitting. Boosted cost-sensitive C5.0 adapts the C5.0 decision tree algorithm by incorporating cost-sensitive considerations, assigning different misclassification costs to minority and majority classes. XGBoost, an ensemble method, employs a gradient boosting framework and is well suited for handling class imbalances through adaptive boosting of weak learners. Adaptive boosting is an artificial intelligence technique that optimizes the performance of an algorithm by using a number of weaker and less efficient algorithms in combination. By combining these weaker algorithms, a more powerful model can be built. The same principle can also be used to combine complex algorithms and improve their accuracy. In addressing imbalanced data, the aim is therefore to minimize the overall misclassification cost and mitigate the problem of imbalanced data. To assess the performance of these algorithms, we used several parameters: AUROC, precision–recall area under curve (PRAUC), balanced accuracy, *F*1 score, sensitivity, specificity, positive and negative prediction values, McNemar test *P*-value, and Brier score. Due to the low occurrence of events and the imbalanced nature of the data, the *F*-score and the precision–recall curve were preferred to determine the best model. We used a five-fold cross-validation process to fit the model hyperparameters using grid search (R package: caret).

To evaluate the interest of the CICU classification model, the performance of the proposed ML model will be compared to a logistic regression with variables selected *a priori* according to their clinical pertinence including age, sex, body mass index, hypertension, diabetes and LVEF, and known scores assessing outcomes in CICU patients from the literature: quick Sequential Organ Failure Assessment (qSOFA), Thrombolysis In Myocardial Infarction (TIMI), Global Registry of Acute Coronary Events (GRACE), and the Acute Heart Failure (ACUTE-HF) score.^18–21^ (see Supplementary material online, *Methods S4*)

### Statistical analysis

Continuous data are reported as mean ± standard deviation for normally distributed data or as medians and interquartile range for non-normally distributed data. Categorical data are reported as counts and percentages. The normality of the variables was verified by histograms, quantile-quantile plot, and Shapiro–Wilk test. Between-group comparisons were made using the Student’s *t*-test or Mann–Whitney test for continuous variables and the χ^2^ or Fisher’s exact test for categorical variables, as appropriate. DeLong’s test was used to compare the AUROC of the ML model and scores used in CICU.^22^

A two-tailed *P*-value <0.05 was considered statistically significant. All data were analysed using R software, version 3.6.3 (R Project for Statistical Computing, R Foundation, Vienna, Austria).

## Results

### Study population

**Figure 1:**
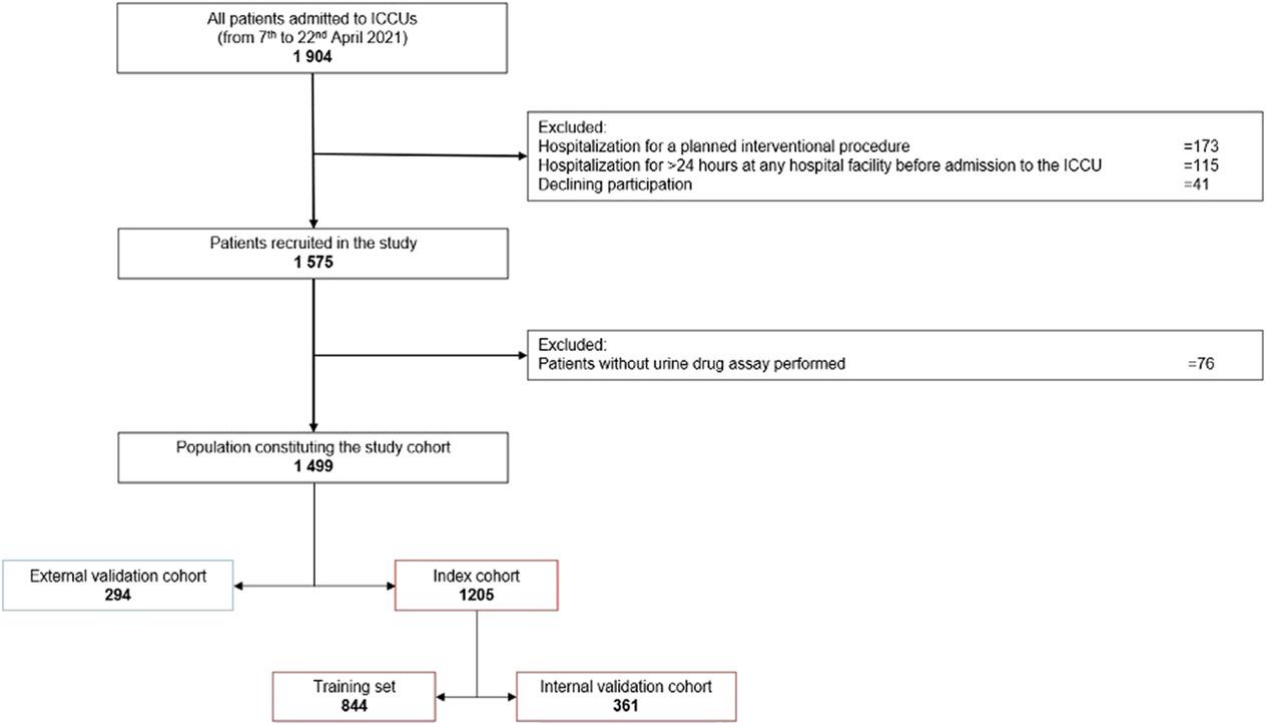
Flow chart of the study population.

Out of 1499 patients included, 1205 (80%) constituted the index cohort, corresponding to the randomly selected 31 centres (*Figure 1*). Patients were mostly men (70.3%) aged 63.5 ± 14.9 years, with one in four being obese (24.4%). In the index cohort, hypertension (53.8%) was the most frequent cardiovascular risk factor followed by dyslipidaemia (39.9%) and smoking (39.5%). Fifty-five patients (4.6%) presented with in-hospital MAE, including 19 (1.6%) in-hospital deaths, 14 (1.2%) cardiac arrest events, and 33 (2.7%) cardiogenic shocks requiring medical and/or mechanical haemodynamic support. Among the 19 in-hospital deaths, 12 patients died due to ventricular arrhythmias identified on the ECG scope after adjudication by an independent centralized committee. Baseline characteristics of subjects according to the onset of MAE are given in *Table 1*.

**Table 1:** Baseline characteristics according to the onset of in-hospital major adverse events in the index cohort (*n* = 1205)

	Index cohort (*n* = 1205)	No MAE (*n* = 1150)	MAE (*n* = 55)	*P*-value
**Demographics**				
Age, years (mean ± SD)	63.5 ± 14.9	63.5 ± 14.7	64.6 ± 17.4	0.64
Men	847 (70.3)	810 (70.4)	37 (67.3)	0.73
BMI, kg/m² (mean ± SD)	27.2 ± 5.5	27.3 ± 5.5	26.2 ± 5.0	0.13
**Cardiovascular risk factors**				
Diabetes	262 (21.7)	248 (21.6)	14 (25.5)	0.61
Hypertension	648 (53.8)	619 (53.8)	29 (52.7)	0.98
Dyslipidaemia	481 (39.9)	462 (40.2)	19 (34.5)	0.49
Current smoker	306 (25.4)	282 (24.5)	24 (43.6)	<0.001
Known CAD	405 (33.6)	387 (33.7)	18 (32.7)	1.0
Family history of CAD	201 (16.7)	198 (17.2)	3 (5.5)	0.04
COPD	51 (4.2)	49 (4.3)	2 (3.6)	1.0
Asthma	14 (1.2)	13 (1.1)	1 (1.8)	0.48
**Medical history of non-cardiovascular disease**				
Cancer	53 (4.40)	49 (4.26)	4 (7.27)	0.19
Psychiatric history	120 (9.96)	112 (9.74)	8 (14.5)	0.35
**Admission diagnosis**				
STEMI	270 (22.4)	254 (22.1)	16 (29.1)	0.29
NSTEMI	355 (29.5)	342 (29.7)	13 (23.6)	0.41
Acute HF	179 (14.9)	163 (14.2)	16 (29.1)	0.004
Arrhythmia	74 (6.1)	72 (6.3)	2 (3.6)	0.57
Myocarditis or pericarditis	56 (4.7)	55 (4.8)	1 (1.8)	0.51
Cardiac conduction abnormalities	65 (5.4)	64 (5.6)	1 (1.8)	0.36
Pulmonary embolism	33 (2.7)	33 (2.9)	0 (0.0)	0.40
Others^a^	173 (14.4)	167 (14.5)	6 (10.9)	0.57
**Clinical parameters at admission**				
Mean arterial pressure, mmHg (mean ± SD)	98 ± 18	99 ± 17	88 ± 20	<0.001
Heart rate, b.p.m. (mean ± SD)	82 ± 24	82 ± 24	89 ± 28	0.08
Oxygen saturation, % (mean ± SD)	97.0 ± 5.6	97.0 ± 5.7	96.9 ± 2.7	0.69
Killip class				<0.001
I	996 (82.7)	964 (83.8)	32 (58.2)	
II	136 (11.3)	124 (10.8)	12 (21.8)	
>III	70 (5.81)	59 (5.13)	11 (20.0)	
**Biological parameters at admission** (mean ± SD)				
Haemoglobin, g/dL	13.5 ± 2.0	13.5 ± 1.9	12.9 ± 2.5	0.08
Creatinaemia, µmol/L	98 ± 72	97 ± 72	111 ± 61	0.11
High-sensitivity cardiac troponin peak, Ul/L [median (IQR)]	11.2 (1.75; 169)	10.6 (1.69; 155)	98.2 (2.96; 644)	0.53
NTproBNP, pg/mL [median (IQR)]	2555 (599; 11 251)	2451 (594; 11 170)	8537 (1007; 15 273)	0.03
**Echocardiographic data**				
LV dilatation ≥ 75 mL/m^2^	114 (9.5)	110 (9.6)	4 (7.3)	0.74
LA dilatation ≥ 32 mL/m^2^	222 (18.4)	201 (17.5)	21 (38.2)	<0.001
Ratio *E*/*A* (mean ± SD)	1.1 ± 0.6	1.1 ± 0.6	1.4 ± 0.6	0.008
Ratio *E*/*e′* > 14	113 (9.4)	99 (8.6)	14 (25.5)	<0.001
Ratio *E*/*e′* (mean ± SD)	9.1 ± 4.1	8.8 ± 3.8	13.3 ± 5.7)	<0.001
LVEF ≤ 25%	82 (6.8)	71 (6.2)	11 (20.0)	<0.001
LVEF (mean ± SD)	52.2 ± 13.2	52.7 ± 12.8	42.1 ± 17.3	<0.001
LVOT VTI (mean ± SD)	19. ± 5	20 ± 5	16 ± 6	<0.001
sPAP (mean ± SD)	35.2 ± 14.1	34.6 ± 13.9	44.6 ± 13.7	<0.001
RV dilatation	79 (6.6)	71 (6.2)	8 (14.5)	0.02
TAPSE ≤ 17 mm	178 (14.8)	166 (14.4)	12 (21.8)	<0.001
TAPSE (mean ± SD)	20.9 ± 4.5	21.0 ± 4.4	17.5 ± 5.3	<0.001

^a^Other admission diagnoses included: coronary spasm, Takotsubo, aortic dissection, spontaneous coronary dissection, chest pain without identified cardiac cause, and other cardiovascular or non-cardiovascular diagnosis.

BMI, body mass index; CAD, coronary artery disease; HF, heart failure; LA, left atrium; LV, left ventricle; LVEF, left ventricular ejection fraction; LVOT VTI, left ventricular outflow tract velocity time integral; NSTEMI, non-ST elevation myocardial infarction; NTproBNP, N-terminal prohormone of brain natriuretic peptide; RV, right ventricle; SD, standard deviation; sPAP, systolic pulmonary artery pressure; STEMI, ST elevation myocardial infarction.

### Feature selection

**Figure 2:**
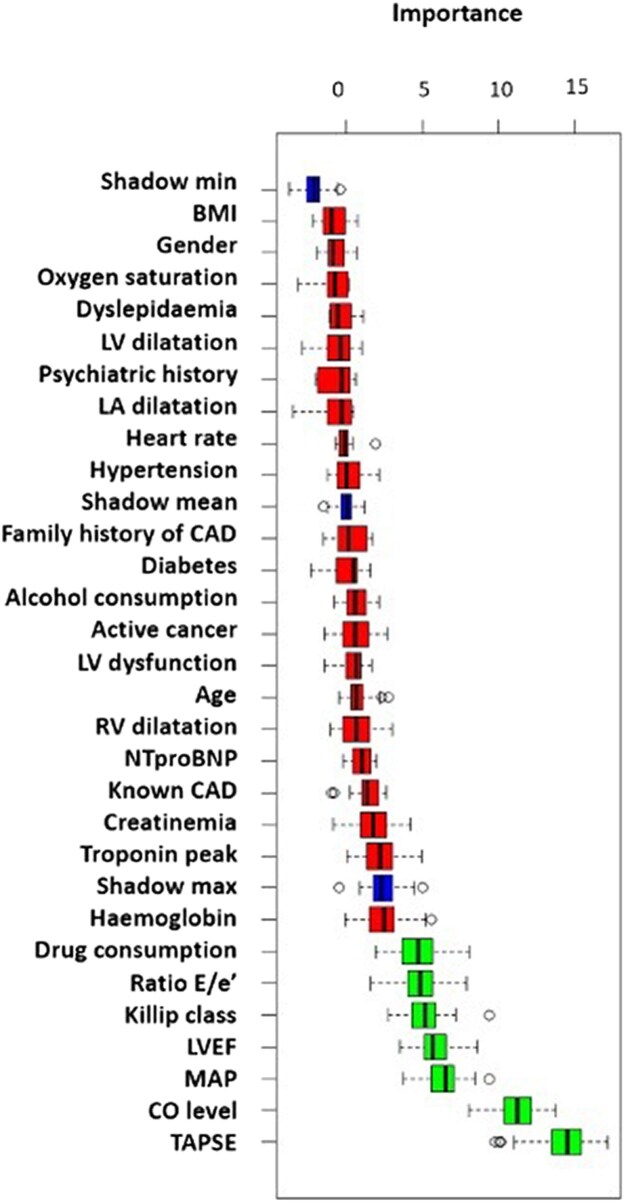
Feature selection using Boruta technique. The Boruta variable selection algorithm was applied to obtain the variables that contribute the most to the prediction of major adverse events. Considering a confidence level with a *P*-value threshold of 0.05, seven variables were considered important: tricuspid annular plane systolic excursion, carbon monoxide level, mean arterial pressure, left ventricular ejection fraction, Killip class, and ratio *E*/*e*′.

Using the Boruta feature selection method, seven of the available variables were selected for the ML model (four clinical and three echocardiographic parameters): illicit drug use, mean arterial pressure, Killip class, exhaled CO level, LVEF, TAPSE value, and peak *E*/*e*′ ratio (*Figure 2*). Tricuspid annular plane systolic excursion value and exhaled CO level were the two highest ranked features for in-hospital MAE prediction (see Supplementary material online, *Figure S1*). No differences were observed in the training and internal validation seta with respect to any of the above seven variables (see Supplementary material online, *Table S4*).

### Prediction of in-hospital major adverse events

**Figure 3:**
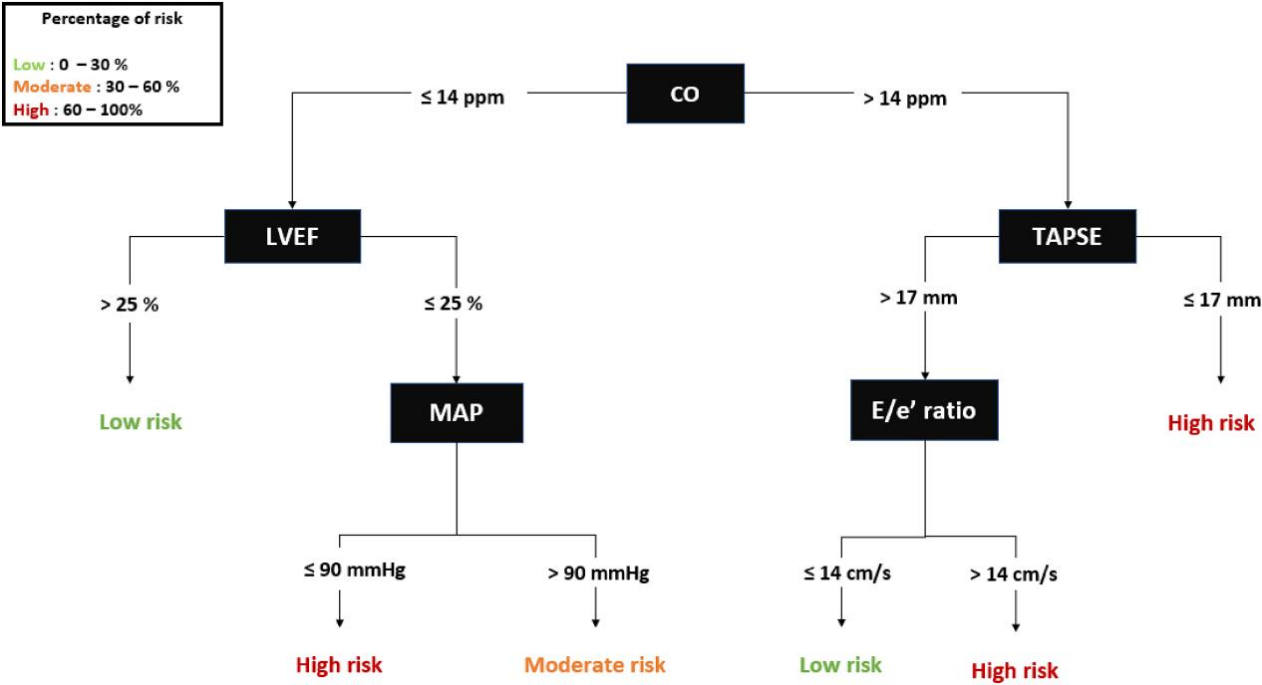
Decision tree illustrating the machine learning model. Boosted C5.0 model is based on the idea of adaptive boosting. The main objective is to combine several weak classifiers into a stronger one, while keeping the robustness to overfitting of the weak classifiers. The classification tree with the lowest error (3.4%) is shown below.

Boosted cost-sensitive C5.0 algorithm showed the AUROC: 0.90 [95% confidence interval (CI): 0.75–0.98] compared with logistic regression (AUROC = 0.84; 95% CI: 0.72–0.96), ridge regression (AUROC:0.85; 95% CI: 0.72–0.96), and XGBoost (AUROC: 0.83; 95% CI: 0.71–0.95) techniques. When assessed using PRAUC or *F*-score, boosted cost-sensitive C5.0 algorithm still showed better performance compared with other ML techniques, as given in *Table 2*. Detailed ROC and PR curves comparing ML techniques and logistic regression are available in Supplementary material online, *Figure S2*. In a sensitivity analysis based on a resampled data set (offsetting the low number of events), boosted cost-sensitive C5.0 algorithm still showed the best performance. (see Supplementary material online, *Figure S3*). The optimal hyperparameters were number of boosting iterations = 20, model type = tree, cost = 1, and no winnow. For illustrative purpose only, the most accurate tree with the lowest margin of error (3.4%) was extracted from the C5.0 model (*Figure 3*). Calibration plot showed good agreement between ML classification and the observed in-hospital risk of MAE (see Supplementary material online, *Figure S4*).

**Table 2:** Supervised learning machine algorithms to predict in-hospital major adverse events

Model evaluation metrics	Logistic regression	Ridge regression	Boosted C5.0	XGBoost
AUROC (95% CI)	0.84 (0.72–0.96)	0.85 (0.72–0.96)	0.90 (0.75–0.98)	0.83 (0.71–0.95)
Precision–recall AUC	0.53	0.54	0.57	0.42
Balanced accuracy	0.66	0.66	0.70	0.62
*F*-score	0.42	0.42	0.50	0.33
Sensitivity	0.33	0.34	0.42	0.25
Specificity	0.98	0.98	0.99	0.99
Positive predictive value	0.57	0.57	0.63	0.50
Negative predictive value	0.97	0.96	0.97	0.96
McNemar test *P*-value	0.23	0.23	0.34	0.15
Brier score	0.91	0.9	0.68	0.95

CO, carbon monoxide; LVEF, ventricular ejection fraction; MAP, mean arterial pressure; TAPSE, Tricuspid annular plane systolic excursion.

**Figure 4:**
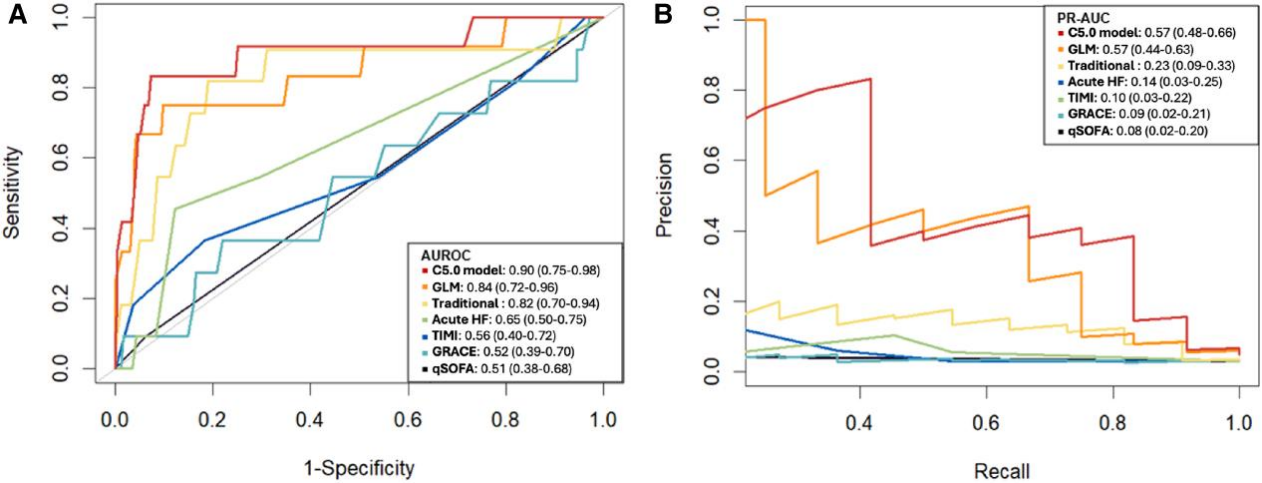
Compared performances of our machine learning model and existing risk scores. (*A*) shows the receiver operating curves and (*B*) shows the precision–recall curves comparing the performances of our machine learning model, logistic regression (including the same variables as the machine learning model), a traditional model (clinical *a priori* regression model), and other existing scores in the internal validation data set of the index cohort. The machine learning model had a significantly higher area under the curve for receiver operating curves and precision–recall curves in predicting in-hospital major adverse events than all other risk models (*P* < 0.001). The results are given with their 95% confidence interval.

Receiver operating characteristic and PR curves comparing the ML model to previously existing CICU scores are shown in *Figure 4*. The prediction performance of the ML model (AUROC: 0.90) was significantly superior to that of the quick Sequential Organ Failure Assessment (qSOFA) (AUROC: 0.51, *P* < 0.001), TIMI (AUROC: 0.56, *P* = 0.005), GRACE (AUROC: 0.52, *P* = 0.001), and ACUTE-HF (AUROC: 0.65, *P* = 0.017) scores. Machine learning model also showed improved performance compared with a traditional logistic regression model (AUROC: 0.90 vs. 0.82, respectively, *P* = 0.032). A sensitivity analysis conducted in the subgroup of patients with HF (*n* = 179) confirmed that the ML model also outperformed ACUTE-HF score (AUROC: 0.89 vs. 0.60, *P* = 0.002 and PRAUC: 0.56 vs. 0.17) (see Supplementary material online, *Figure S5*). Similar results were found in the ACS subgroup (*n* = 625): ML model showed improved prediction performance compared with TIMI and GRACE scores (AUROC: 0.94 vs. 0.51 and 0.64, respectively, *P* < 0.001 and PRAUC: 0.61 vs. 0.22 and 0.17, respectively) (see Supplementary material online, *Figure S6*)

No difference was found according to the admission in university vs. non-university hospitals (AUROC: 0.88 vs. 0.91, respectively, *P* = 0.08; and PRAUC: 0.55 vs. 0.58, respectively, *P* = 0.07).

### Validation of the machine learning model in the independent cohort

Out of 294 patients included in eight independent centres, 12 (4.1%) presented MAE, with no significant difference compared with the index cohort (4.6% of in-hospital MAE, *P* = 0.84). The independent validation cohort was similar to the index cohort regarding demographics (66.7% men, aged 62.2 ± 15.1 years), cardiovascular risk factors, and medical history. Characteristics of the independent validation cohort compared with the index cohort are detailed in Supplementary material online, *Table S5*.

Our proposed ML model, assessed in the independent validation cohort, presented similar AUROC compared with the index cohort (AUC: 0.88 vs. 0.90, respectively). Consistently, the ML model exhibited a higher AUROC in the independent cohort compared with qSOFA, TIMI, GRACE, ACUTE-HF score, and a traditional logistic regression model (ML: 0.88 vs. qSOFA: 0.50, TIMI: 0.73, GRACE: 0.71, ACUTE-HF: 0.61, logistic regression: 0.86). Similar results were found regarding PRAUC (see Supplementary material online, *Figure S7*).

## Discussion

Using ML techniques in a prospective multicentre cohort study of consecutive patients admitted to CICU, we trained a model to predict in-hospital outcome. This ML model was based on few simple clinical and echocardiographic variables: illicit drug use, mean arterial pressure, Killip class, exhaled CO level, LVEF, TAPSE value, and peak *E*/e′ ratio. It showed greater prognostic value to predict in-hospital outcomes than several traditional scores, including qSOFA, TIMI, GRACE, and ACUTE-HF, with similar performance in an external validation cohort.

In our study, we reported a rate of in-hospital MAE of 4.6% and in-hospital death of 1.6%. Prior American and Canadian studies have shown a higher death rate estimated around 8.5% in overall patients hospitalized in CICU.^23,24^ In parallel, studies conducted in coronary intensive care unit including mainly ACSs showed death rates closer to our study around 4.5%.^25^ However, we observed baseline demographic features highly similar to that of prior CICU populations with a vast majority of men and a mean age around 65 years.^23,26^ This low rate of in-hospital death in our study could then be related to the high proportion of ACSs in the ADDICT-CICU trial. It has been shown that death and adverse events among patients hospitalized for ACS have decreased over the past decades and remain lower than that of patients admitted for acute HF.^26,27^

Exhaled CO was one of the highest ranked features for risk prediction in our analysis. It has been previously described as a measure of particular interest for the study of tobacco exposure and is used in the monitoring of smoking cessation.^14^ A subanalysis of patients from the Framingham cohort showed an association between exhaled CO and the occurrence of cardiovascular disease or metabolic syndrome, independent of smoking status.^28^ Our working group has already shown that exhaled CO, not only associated with the risk of developing cardiovascular disease, would also be associated with the in-hospital prognosis in the acute phase once the cardiovascular disease is declared.^10,29^ This finding is also supported by Tun *et al*.^30^ who showed that increased levels of exhaled CO were associated with an adverse cardiovascular biomarker profile and a higher risk of HF, especially with reduced ejection fraction. Exhaled CO may therefore be an interesting predictive marker for acute HF as well as coronary artery disease through its association with a poor cardiovascular risk profile.

Illicit drug use was also integrated as an important variable to build our ML model. This finding is in line with a prior analysis from ADDICT-CICU study showing that illicit drug use is strongly associated with the occurrence of in-hospital outcomes with an incremental prognostic value above traditional risk factors.^29^ These findings can be explained by several types of sympathomimetic effects of illicit drugs which can increase blood pressure, heart rate, temperature, and consequently myocardial oxygen demand.^31^

We observed a strong contribution of echocardiographic parameters to risk prediction. The three echocardiographic parameters retained in the variables of importance reflected left, right, and diastolic ventricular function with LVEF, TAPSE index, and *E*/*e′* ratio. Many studies have shown that left ventricular dysfunction, evaluated using global longitudinal strain or LVEF, was associated with a poor prognosis in a wide spectrum of cardiovascular diseases.^32,33^ Right ventricular dysfunction in the acute setting of coronary syndrome and HF has also been described as an important prognostic factor.^34,35^ The same is true regarding prognostic significance of filling pressure, which was well known in HF and has recently been demonstrated in the field of ACSs.^36^ These findings are in line with a recent study which showed a specific association of the *E*/*e*’ ratio with overall in-hospital death in CICU.^37^

Our ML model exhibited improved prognostic value to predict in-hospital outcomes than several traditional scores assessed in CICU, including traditional logistic regression. Our ML model included only seven variables to outstripped GRACE and TIMI scores in ACS, and ACUTE-HF score in acute HF.^19,20^ These good performance could be partly explained by the many-faceted nature of the model: each variable captures a different facet of the patient’s medical evaluation (i.e. haemodynamic, inotropism, fluid overload) that could have consequences on both the short and medium term. Besides, the small number of variables ensures a practical and easy use in everyday practice. Up to date, no specific ML model including clinical, biological, and echocardiographic data has been built.^38^ The Mayo Clinic team recently developed a clinicobiological risk model that does not include echocardiographic and ECG characteristics. The M-CICU Admission Risk Model (M-CARS) was also developed specifically for CICU patients.^39^ The M-CARS model consists of seven variables, one of which is the Braden skin model and three are biological results (blood urea nitrogen, anion gap, and red blood cell distribution width). Unfortunately, we did not have all the data necessary to calculate this model and could not compare the performance of our ML model with that of M-CARS. Most of the scores developed to date have produced good results using traditional statistical methods such as logistic regression. Although our results show a better performance of the ML model, some data shade the interest of ML techniques in the prediction of medical risk. Large systematic reviews suggest that, in the presence of bias, poor calibration, and/or other methodological limitations, ML often has no performance advantage over logistic regression models.^40^ Larger external validation cohorts in other regions or other healthcare systems are needed to confirm the performance of the ML model.

Interestingly, the ML model seems to distinguish two groups of patients. On the one hand, right ventricular function of smokers is of particular interest. This result may be explained by the association of smoking with an increased incidence of respiratory diseases such as obstructive lung disease or elevated pulmonary pressures. The tree then differentiates the patients in this group as having or not having diastolic dysfunction, illustrating the possible evolution of right heart pathology on left pressures. Non-smoking patients, on the other hand, have a greater burden of HF and haemodynamic signs, with a thorough evaluation of signs of cardiogenic (pre-)shock. These results demonstrate other interests and advantages of ML techniques based on decision trees. In fact, they can also be used to identify subgroups of patients with different risk profiles. Here, the subgroups identified by the ML model correspond to genuine patient populations with their own characteristics.

### Study limitations

We acknowledge some limitations. First, the sample of patients analysed was reduced after splitting between training and validation cohorts. In addition, the exclusion of eight centres to build an independent validation cohort also lowered the sample size. This implies that the subgroup of patients with the same hospitalization reason, such as HF or ACS, also had small sample size. The performance of this ML score on these diagnostic subcategories should therefore be interpreted cautiously. Nevertheless, as there is no other study on the CICU population that has measured exhaled CO and illicit drug use, the results necessarily had to be validated on the ADDICT-CICU cohort. Indeed, we acknowledge as a significant limitation the lack of external validation of our ML model outside of France. Second, although we observed a small number of in-hospital events, we adapted our statistical analysis with algorithm and resampling techniques to deal with unbalanced data.^41^ A specific post hoc sensitivity analysis was carried out for this purpose. Although the high heterogeneity of the population of this study is a significant challenge and a limitation of the analysis, it is important to highlight similar performances of our ML score in the subgroup analyses performed. Third, we noted missing data on ultrasound variables, up to 25% for the *E*/*e*′ ratio, related to the feasibility of the examination in the acute phase. Although echocardiography is carried out at the patient’s bedside, it is not always easy to perform within the first 24 h of admission in many centres around the world. Still, the selection of variables was performed only on patients without missing data and a post hoc sensitivity analysis conducted in overall patients showed similar results. Fourth, our final model included illicit drug use and exhaled CO level, which are not routinely captured in all CICUs. However, a growing body of evidence points to the value of systematically assessing drug use at CICU admission, and exhaled CO levels can help assess respiratory status. We also collected information on illicit drug use at the time of admission to hospital and on history of drug use, but we can neither establish nor rule out a causal link between drug use and hospitalization. In addition, some biological data such as lactate dosage or interventional findings such as the frequency of the need for ventilation, organ replacement procedures, and/or SAPS/TISS were not systematically performed in the patients of this study due to the initial haemodynamic stability at baseline. Fifth, although this was a multicentre study, it was not exhaustively conducted in all French centres. Yet, the 39 centres that participated in the study included centres in large metropolitan areas, medium-sized cities, public university hospitals, non-university hospitals, and private clinics, ensuring a fairly representative sample of French CICUs.

## Conclusion

Using data from a prospective multicentre cohort study, we built a ML model to predict in-hospital outcomes of patients admitted to CICU. Based on few simple clinical and echocardiographic variables (illicit drug use, mean arterial pressure, Killip class, exhaled CO level, LVEF, TAPSE value, and peak *E*/*e*′ ratio) collected at admission, this ML model showed improved performance to predict in-hospital outcomes than several traditional scores, including the qSOFA, TIMI, GRACE, and ACUTE-HF scores. Finally, this ML score has been designed to be quick and easily feasible to facilitate early triage and target patients at high risk of clinical worsening.

## Supplementary Material

ztae098_Supplementary_Data

## Data Availability

Data can be available upon request to the corresponding author.
