# Supplementary material for: Machine learning score to predict in-hospital outcomes in patients hospitalized in cardiac intensive care unit
Source: Eur Heart J Digit Health. 2024 Dec 20;6(2):218–27. doi: 10.1093/ehjdh/ztae098 (PMC11914730; doi:10.1093/ehjdh/ztae098)
Supplement: ztae098_Supplementary_Data [file ztae098_supplementary_data.docx]

**Supplementary Appendix**

Contents

[eMethods 2: Reliability parameters of the urine drug assay 9](#_Toc178860043)

[eMethods 3: Echocardiographic parameters collected in the ADDICT-ICCU study 10](#_Toc178860044)

[eMethods 4: Variables included in existing scores calculated in the study. 11](#_Toc178860045)

[eFigure 1: Feature importance in the machine learning model. 12](#_Toc178860046)

[eFigure 2: Compared performances of machine learning techniques and logistic regression in internal validation dataset of the index cohort. 13](#_Toc178860047)

[eFigure 3. Compared performances of machine learning techniques and logistic regression in resampled data sets. 14](#_Toc178860048)

[eFigure 4: Calibration of the machine learning model in the index cohort. 15](#_Toc178860049)

[eFigure 5: Compared performances of our machine learning model and existing risk scores among patients admitted for heart failure in the index cohort (N = 179) 16](#_Toc178860050)

[eFigure 6. Compared performances of our machine learning model and existing risk scores among patients admitted for acute coronary syndrome in the index cohort (N = 625) 17](#_Toc178860051)

[eFigure 7. Compared performances of our machine learning model and existing risk scores in the external validation cohort (N = 294) 18](#_Toc178860052)

[eTable 1: Participating centres, number of patients enrolled and belonging cohort. 19](#_Toc178860053)

[eTable 2. Variables used in the feature selection algorithm. 21](#_Toc178860054)

[eTable 3. Missing data in the final machine learning model 22](#_Toc178860055)

[eTable 4. Compared selected features of the machine learning model in the training and internal validation sets within the index cohorts 23](#_Toc178860056)

[eTable 5. Compared characteristics of the external derivation cohort and index cohort. 24](#_Toc178860057)

*eMethods 1*: ADDICT-ICCU Investigators (alphabetical order)

| First name | Last name | Center |
| --- | --- | --- |
| Victor | ABOYANS | Department of Cardiology, University Hospital of Limoges, France. |
| Emeric | ALBERT | Department of Cardiology, Hôpital Européen Georges Pompidou (HEGP), France. |
| Franck | ALBERT | Service de Cardiologie, Centre Hospitalier de Chartres, 28630 Le Coudray, France. |
| Sean | ALVAIN | Service de Cardiologie, Centre hospitalier de Saintonge, 11, boulevard Ambroise-Paré, 17100 Saintes, France. |
| Nabil | AMRI | Service de Cardiologie Interventionnelle, CHU Timone, APHM, Aix Marseille Univ, Marseille, France |
| Stéphane | ANDRIEU | Service de cardiologie, Hôpital Henri Duffaut, 84902 AVIGNON, France |
| Sabir | ATTOU | Department of Cardiology, Caen University Hospital, Caen, France |
| Simon | AUVRAY | Department of Cardiology, Felix-Guyon University Hospital, Saint-Denis-de-La-Reunion, France |
| Sonia | AZZAKANI | Department of Cardiology, University Hospital of Poitiers, 86000 Poitiers, France. |
| Ruben | AZENCOT | Service de Cardiologie, Hôpital Cochin, Paris, France |
| Marc | BEDOSSA | CHU Rennes, Service de Cardiologie et Maladies Vasculaires, Rennes, F-35000, France |
| Franck | BOCCARA | Department of Cardiology, Saint-Antoine Hospital, APHP, Sorbonne University, Paris, France |
| Albert | BOCCARA | Department of Cardiology Andre Gregoire Hospital 93100 Montreuil, France |
| Thomas | BOCHATON | Intensive Cardiological Care Division, Louis Pradel Hospital, Hospices Civils de Lyon, Bron, France. |
| Eric | BONNEFOY-CUDRAZ | Intensive Cardiological Care Division, Louis Pradel Hospital, Hospices Civils de Lyon, Bron, France. |
| Guillaume | BONNET | Service de Cardiologie Interventionnelle, CHU Timone, APHM, Aix Marseille Univ, INSERM, INRAE, C2VN, Marseille, France |
| Guillaume | BONNET | Hôpital Cardiologique Haut-Lévêque, Centre Hospitalier Universitaire de Bordeaux, Unité médico-chirurgical de valvulopathies et cardiomyopathies, 33600 Pessac, France. |
| Nabil | BOUALI | Service de Cardiologie, Centre hospitalier de Saintonge, 11, boulevard Ambroise-Paré, 17100 Saintes, France. |
| Océane | BOUCHOT | Service de Cardiologie, Centre hospitalier Annecy Genevois, 1 Avenue de l'Hôpital, 74370 Epagny Metz-Tessy |
| Claire | BOULETI | Department of Cardiology, University Hospital of Poitiers, 86000 Poitiers, France. |
| Tanissia | BOUKERTOUTA | Department of Cardiology, Hôpital Avicenne, Assistance Publique-Hôpitaux de Paris, Paris, France |
| Jean Baptiste | BRETTE | Cardiology department, Rangueil University Hospital, Toulouse, France |
| Marjorie | CANU | Service de Cardiologie, CHU Grenoble-Alpes, France. |
| Aures | CHAIB | Department of Cardiology Andre Gregoire Hospital 93100 Montreuil, France. |
| Clement | CHARBONNEL | Service de Cardiologie, Hôpital Mignot, Versailles, France. |
| Anne Solene | CHAUSSADE | Clinique A Paré, Neuilly/seine, département de Cardiolgie. |
| Alexandre | COPPENS | Department of Cardiology Andre Gregoire Hospital 93100 Montreuil, France |
| Yves | COTTIN | Department of Cardiology, University Hospital, Dijon, France. |
| Arthur | DARMON | Université de Paris, Department of Cardiology, Hôpital Bichat, Assistance Publique-Hôpitaux de Paris, Paris, France. |
| Elena | DE ANGELIS | Intensive Cardiological Care Division, Louis Pradel Hospital, Hospices Civils de Lyon, Bron, France. |
| Clément | DELMAS | Intensive Cardiac Care Unit, Rangueil University Hospital, Toulouse, France. |
| Laura | DELSARTE | Department of Cardiology, University Hospital of Brest, 29609, Brest cedex, France. |
| Antoine | DENEY | Cardiology department, Rangueil University Hospital, Toulouse, France |
| Jean Claude | DIB | Clinique A Paré, Neuilly/seine, département de Cardiolgie, France. |
| Jean-Guillaume | DILLINGER | Université de Paris, Department of Cardiology, Hôpital Lariboisière, Assistance Publique-Hôpitaux de Paris, Inserm U-942, Paris, France |
| Clemence | DOCQ | Department of Cardiology, University Hospital of Lille, France. |
| Valentin | DUPASQUIER | Department of Cardiology, CHU Montpellier, France. |
| Meyer | ELBAZ | Cardiology department, Rangueil University Hospital, Toulouse, France |
| Antony | EL HADAD | Service de cardiologie - Hôpital MONTFERMEIL 93370, France. |
| Amine | EL OUAHIDI | Department of Cardiology, University Hospital of Brest, 29609, Brest cedex, France |
| Nacim | EZZOUHAIRI | Cardiology Intensive Care Unit and Interventional Cardiology, Hôpital Cardiologique du Haut-Lévêque, Pessac, France |
| Julien | FABRE | Department of Cardiology, University Hospital of Martinique, France. |
| Damien | FARD | Intensive Cardiac Care Unit University Hospital Henri Mondor Créteil France |
| Charles | FAUVEL | Department of Cardiology, Rouen University Hospital, 76000 Rouen, France |
| Édouard | GERBAUD | Cardiology Intensive Care Unit and Interventional Cardiology, Hôpital Cardiologique du Haut-Lévêque, Pessac, France et Bordeaux Cardio-Thoracic Research Centre, U1045, Bordeaux University, Bordeaux, France |
| Martine | GILARD | Department of Cardiology, University Hospital of Brest, 29609, Brest cedex, France |
| Marc | GORALSKI | Service de Cardiologie, Centre Hospitalier d’Orleans, Orléans, France |
| Nissim | GRINBERG | Service de Cardiologie, Hôpital Mignot, Versailles, France. |
| Alain | GRENTZINGER | Service de Cardiologie, Centre hospitalier de Saintonge, 11, boulevard Ambroise-Paré, 17100 Saintes, France. |
| Marie | HAUGUEL-MOREAU | Service de Cardiologie, Boulogne Billancourt, Hôpital Ambroise Pare, University Hospital Center, AP-HP. |
| Patrick | HENRY | Université de Paris, Department of Cardiology, Hôpital Lariboisière, Assistance Publique-Hôpitaux de Paris, Inserm U-942, Paris, France |
| Fabien | HUET | Department of Cardiology, CHU Montpellier, France. |
| Thomas | LANDEMAINE | Unité de Soins intensifs Cardiologiques, CHU Amiens, 80000 Amiens, France |
| Benoit | LATTUCA | Department of Cardiology, Nîmes University Hospital, Montpellier University, Nîmes, France |
| Léo | LEMARCHAND | CHU Rennes, Service de Cardiologie et Maladies Vasculaires, Rennes, F-35000, France |
| Thomas | LEVASSEUR | Service de Cardiologie, centre hospitalier de Fréjus/Saint-Raphaël, 83600 Fréjus, France. |
| Pascal | LIM | Intensive Cardiac Care Unit University Hospital Henri Mondor Créteil France |
| Laura | MAITRE BALLESTEROS | Service de Cardiologie, CHU Grenoble-Alpes, France. |
| Nicolas | MANSENCAL | Service de Cardiologie, Boulogne Billancourt, Hôpital Ambroise Pare, University Hospital Center, AP-HP |
| Benjamin | MARIE | Cardiology Intensive Care Unit and Interventional Cardiology, Hôpital Cardiologique du Haut-Lévêque, Pessac, France |
| David | MARTINEZ | Department of Cardiology, Nîmes University Hospital, Montpellier University, Nîmes, France |
| Benoit | MERAT | Service de cardiologie et médecine aéronautique, Hôpital d'Instruction des Armées Percy, 101 avenue Henri Barbusse, 92140 Clamart, France. |
| Christophe | MEUNE | Department of Cardiology, Hôpital Avicenne, Assistance Publique-Hôpitaux de Paris, Paris, France. |
| Damien | MILLISCHER | Service de cardiologie - Hôpital MONTFERMEIL 93370, France. |
| Thomas | MOINE | NCT+, Saint Cyr Sur Loire, France |
| Pascal | NHAN | Department of Cardiology, Saint-Antoine Hospital, APHP, Sorbonne University, Paris, France |
| Nathalie | NOIRCLERC | Service de Cardiologie, Centre hospitalier Annecy Genevois, 1 Avenue de l'Hôpital, 74370 Epagny Metz-Tessy |
| Patrick | OHLMANN | Department of Cardiovascular Medicine, Nouvel Hôpital Civil, Strasbourg University Hospital, Strasbourg, France |
| Théo | PEZEL | Université de Paris, Department of Cardiology, Hôpital Lariboisière, Assistance Publique-Hôpitaux de Paris, Inserm U-942, Paris, France |
| Fabien | PICARD | Service de Cardiologie, Hôpital Cochin, Paris, France |
| Nicolas | PILIERO | Service de Cardiologie, CHU Grenoble-Alpes, France |
| Thibaut | POMMIER | Department of Cardiology, University Hospital, Dijon, France. |
| Etienne | PUYMIRAT | Department of Cardiology, Hôpital Européen Georges Pompidou (HEGP), France. |
| Arthur | RAMONATXO | Department of Cardiology, University Hospital of Poitiers, 86000 Poitiers, France. |
| Reza | ROSSANALY VASRAM | Department of Cardiology, Felix-Guyon University Hospital, Saint-Denis-de-La-Reunion, France |
| François | ROUBILLE | Department of Cardiology, CHU Montpellier, France. |
| Vincent | ROULE | Department of Cardiology, Caen University Hospital, Caen, France |
| Guillaume | SCHURTZ | Department of Cardiology, University Hospital of Lille, France. |
| Mathilde | STEVENARD | Service de cardiologie et médecine aéronautique, Hôpital d'Instruction des Armées Percy, 101 avenue Henri Barbusse, 92140 Clamart |
| David | SULMAN | Université de Paris, Department of Cardiology, Hôpital Bichat, Assistance Publique-Hôpitaux de Paris, Paris, France. |
| Fédérico | SWEDSKY | Service de cardiologie, Hôpital Henri Duffaut, 84902 AVIGNON, France |
| Victoria | TEA | Department of Cardiology, Hôpital Européen Georges Pompidou (HEGP), France. |
| Eugénie | THEVENET | Department of Cardiology, University Hospital of Martinique, France. |
| Christophe | THUAIRE | Service de Cardiologie, Centre Hospitalier de Chartres, 28630 Le Coudray, France. |
| Antonin | TRIMAILLE | Department of Cardiovascular Medicine, Nouvel Hôpital Civil, Strasbourg University Hospital, 67000 Strasbourg, France |
| Christophe | TRON | Department of Cardiology, Rouen University Hospital, 76000 Rouen, France |
| Guillaume | VIBOUD | Unité de Soins intensifs Cardiologiques, CHU Amiens, 80000 Amiens, France |
| Dominique | YOMI | Service de Cardiologie, centre hospitalier de Fréjus/Saint-Raphaël, 83600 Fréjus, France. |
| Cyril | ZAKINE | Tours, Clinique Saint Gatien Alliance (NCT+), Saint-Cyr-sur-Loire |

# *eMethods 2*: Reliability parameters of the urine drug assay

To assess the reliability of the NarcoCheck^®^ urine drug assay, a comparative analysis between NarcoCheck^®^ and the findings of the regional reference Laboratory in Biological Toxicology was performed on a random sample of 60 patients. NarcoCheck^®^ urine drug assay had a sensitivity of 91.7% and specificity of 97.9%.

|  | | Reference Laboratory in Biological Toxicology | |
| --- | --- | --- | --- |
|  |  | Positive | Negative |
| NarcoCheck^®^ urine drug assay | Positive | 11 | 1 |
|  | Negative | 1 | 47 |

# *eMethods 3: Echocardiographic parameters collected in the ADDICT-ICCU study*

| Transthoracic echocardiography variables |
| --- |
| Left ventricle and atrium |
| Left ventricular ejection fraction (%) |
| Left ventricular end-diastolic volume indexed to body surface area (mL/m^2^) |
| Left atrial volume indexed to body surface area (mL/m^2^) |
| Maximum end-diastolic LV wall thickness (mm) |
| Mitral E wave (cm/s) |
| Mitral A wave (cm/s) |
| Mitral annulus e' wave (cm/s) |
| Left ventricular outflow track velocity-time integral (cm) |
| Right ventricle and atrium |
| Presence of RV dilatation defined by an RV/LV ratio > 1 measured using the four-chamber view |
| Tricuspid annular plane systolic excursion (mm) |
| Tricuspid regurgitation peak velocity (m/s) |
| Systolic pulmonary arterial pressure (mmHg) |
| Valvular features |
| Presence of significant valvular disease: |
| Aortic regurgitation, defined by grade 2+ |
| Mitral regurgitation, defined by grade 2+ |
| Aortic stenosis, defined by maximum velocity at peak > 3 m/s or mean gradient > 25 mmHg |
| Mitral stenosis, defined by mean gradient > 5 mmHg |
| Tricuspid regurgitation, defined by grade 2+ |
| Presence of endocarditis, according to the modified Duke's criteria, defined by vegetation, valvular perforation or aneurysm, abscess, pseudoaneurysm, intracardiac fistula or new partial dehiscence of prosthetic valve |
| Pericardium |
| Presence of pericardial effusion, defined by > 5 mm thick measured in end-diastole |
| Others |
| Evaluation of the inferior vena cava |
|  |

# *eMethods 4: Variables included in existing scores calculated in the study.*

| Scores | Variables included |
| --- | --- |
| qSOFA ^1^ | - Glasgow coma scale (1-15) - Respiratory rate (cycles per minute) - Systolic blood pressure (mmHg) |
| TIMI ^2^ | - Age (years) - Number of cardiovascular risk factors (≥ 3) - Prior significant coronary artery disease - ST segment elevation on electrocardiogram - Chest pain - Elevated serum cardiac biomarkers - Aspirin use |
| GRACE ^3^ | - Age (years) - Heart rate (beats per minute) - Systolic blood pressure (mmHg) - Serum creatinine (mg/dL) - History of heart failure or myocardial infarction - ST segment depression - Elevated serum cardiac biomarkers - No in-hospital percutaneous coronary intervention done |
| ACUTE-HF ^4^ | - Age (years) - Serum creatinine (mg/dL) - Use of non-invasive ventilation - Stroke or transient ischemic attack - Left ventricular ejection fraction <30% - Prior hospitalization for acute heart failure - Mitral dysfunction |

References:

1. Singer M, Deutschman CS, Seymour CW, et al. The Third International Consensus Definitions for Sepsis and Septic Shock (Sepsis-3). JAMA 2016;315(8):801–10.

2. Antman EM, Cohen M, Bernink PJLM, et al. The TIMI Risk Score for Unstable Angina/Non–ST Elevation MI: A Method for Prognostication and Therapeutic Decision Making. JAMA 2000;284(7):835.

3. Fox K a. A, Eagle KA, Gore JM, Steg PG, Anderson FA, GRACE and GRACE2 Investigators. The Global Registry of Acute Coronary Events, 1999 to 2009--GRACE. Heart 2010;96(14):1095–101.

4. Cameli M, Pastore MC, De Carli G, et al. ACUTE HF score, a multiparametric prognostic tool for acute heart failure: A real-life study. International Journal of Cardiology 2019;296:103–8.

# *eFigure 1*: Feature importance in the machine learning model.


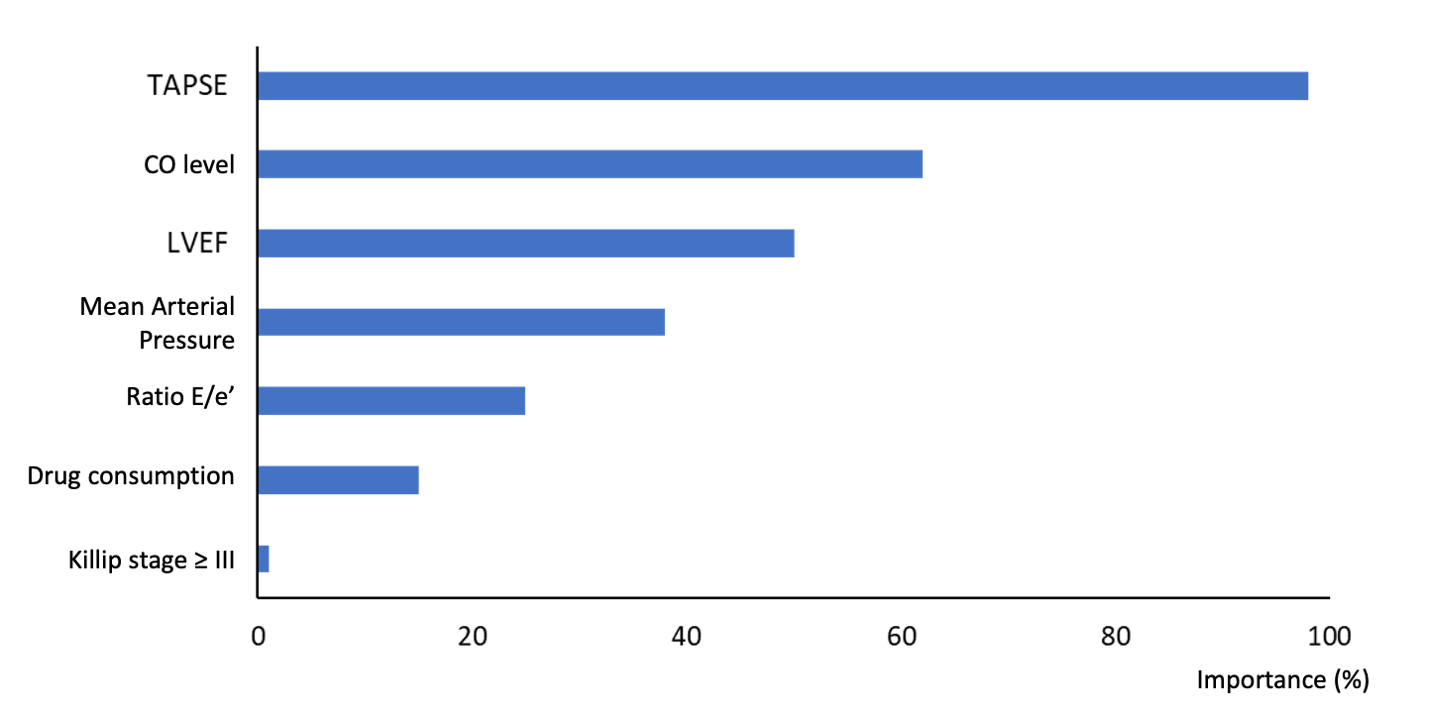


*Abbreviations: CO: Carbon Monoxide, LVEF: Left Ventricle Ejection Fraction, TAPSE: Tricuspid Annular Plane Systolic Excursion.*

*eFigure 2*: Compared performances of machine learning techniques and logistic regression in internal validation dataset of the index cohort.

Panel A shows the Receiver Operating Curves and panel B shows the Precision-Recall Curves comparing the performances of several machine learning techniques and traditional logistic regression. Boosted cost-sensitive C5.0 displayed the best performance.

*
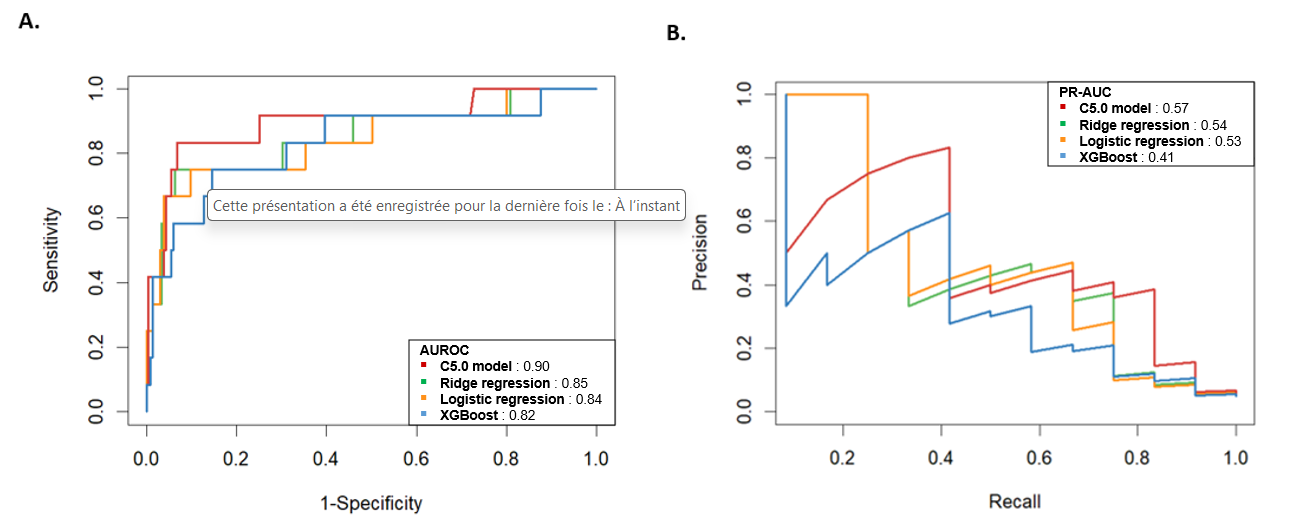
*

*eFigure 3.* Compared performances of machine learning techniques and logistic regression in resampled data sets.

Panel A shows the Receiver Operating Curves and panel B shows the Precision-Recall Curves comparing the performances of several machine learning techniques and traditional logistic regression. Boosted cost-sensitive C5.0 displayed the best performance. Of note, we performed 10 re-sampled data and propose 95% confidence intervals.

*
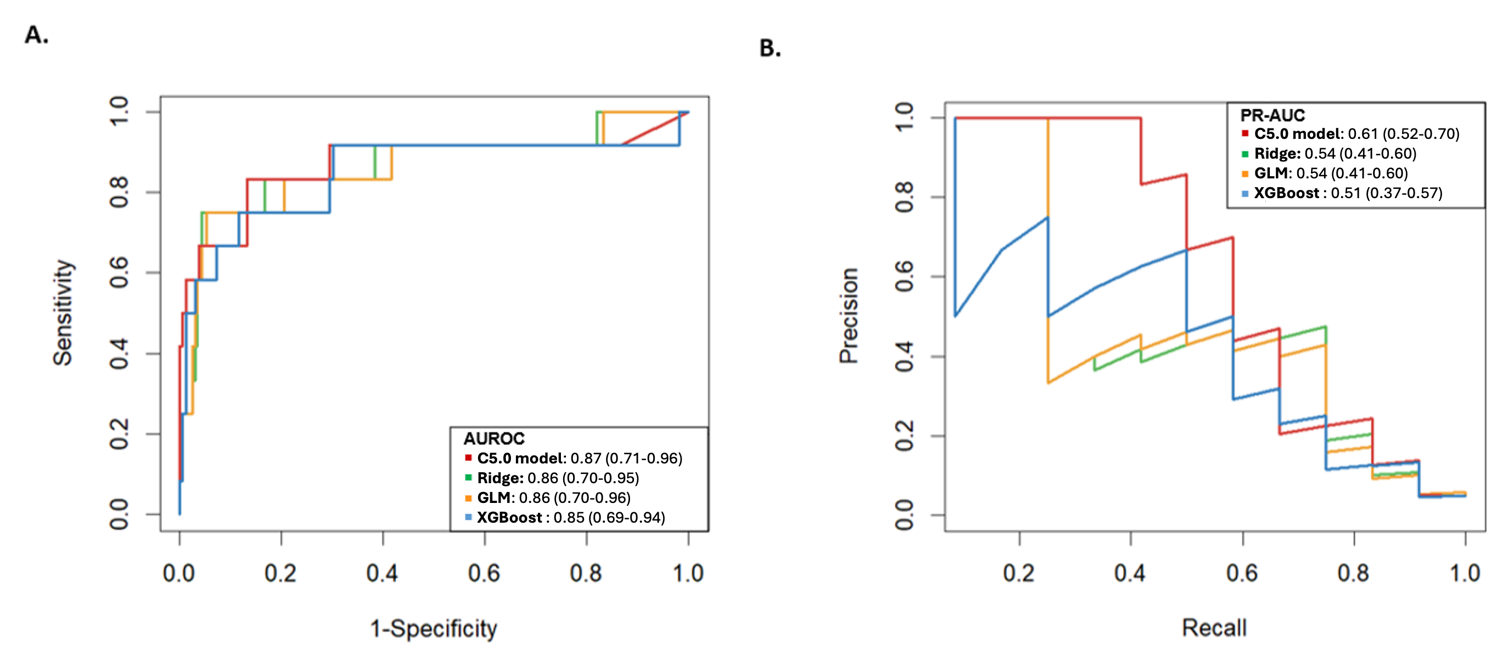
*

# *eFigure 4*: Calibration of the machine learning model in the index cohort.

Due to the small number of MAE, expected events were divided in risk quartile rather than risk deciles.


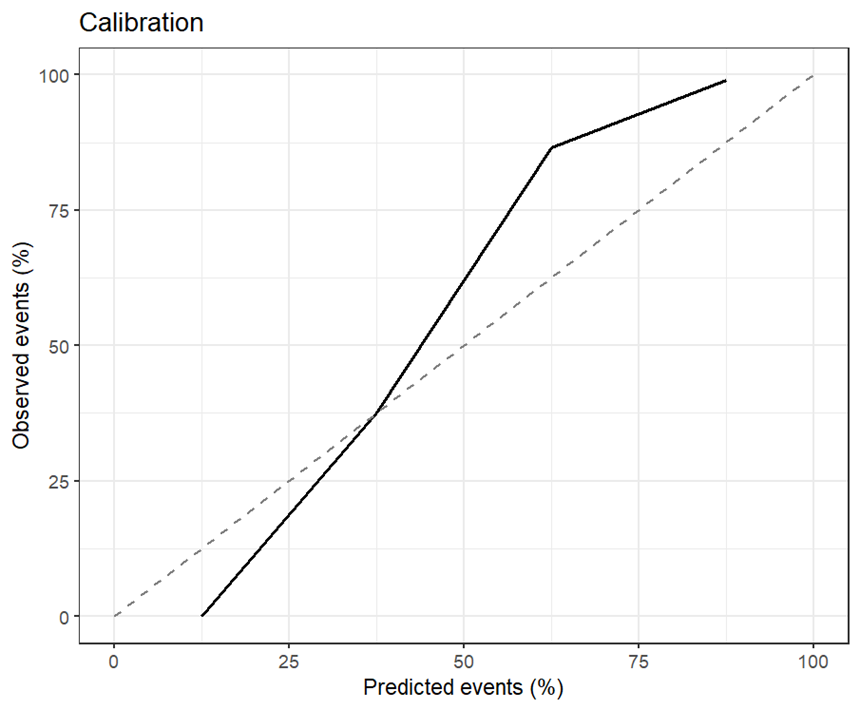


*eFigure 5*: Compared performances of our machine learning model and existing risk scores among patients admitted for heart failure in the index cohort (N = 179)

Panel A shows the Receiver Operating Curves and panel B shows the Precision-Recall Curves comparing the performances of our machine learning model (ML model), logistic regression (including the same variables as the machine learning model), a traditional model (stepwise logistic regression model) and the ACUTE-HF existing score.


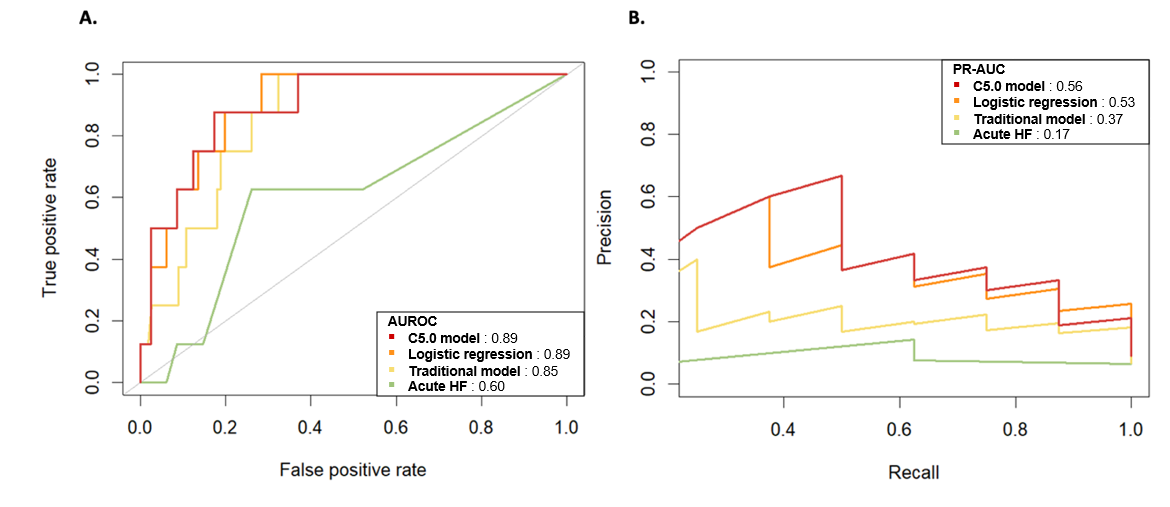


*eFigure 6.* Compared performances of our machine learning model and existing risk scores among patients admitted for acute coronary syndrome in the index cohort (N = 625)

Panel A shows the Receiver Operating Curves and panel B shows the Precision-Recall Curves comparing the performances of our machine learning model (ML model), logistic regression (including the same variables as the machine learning model), a traditional model (stepwise logistic regression model) and two existing scores (TIMI and GRACE scores).


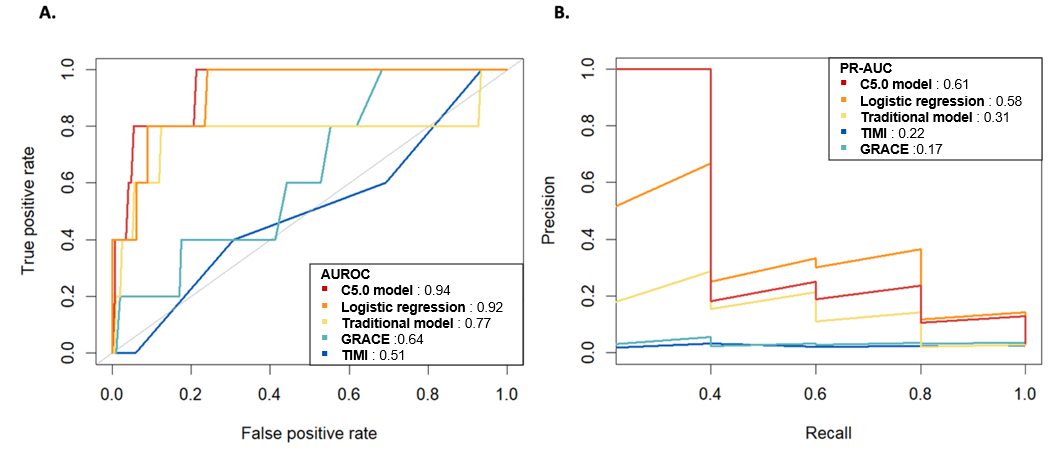


*eFigure 7*. Compared performances of our machine learning model and existing risk scores in the external validation cohort (N = 294)

Panel A shows the Receiver Operating Curves and panel B shows the Precision-Recall Curves comparing the performances of our machine learning model (ML model), logistic regression (including the same variables as the machine learning model), a traditional model (stepwise logistic regression model) and other existing scores in the external validation cohort.

*
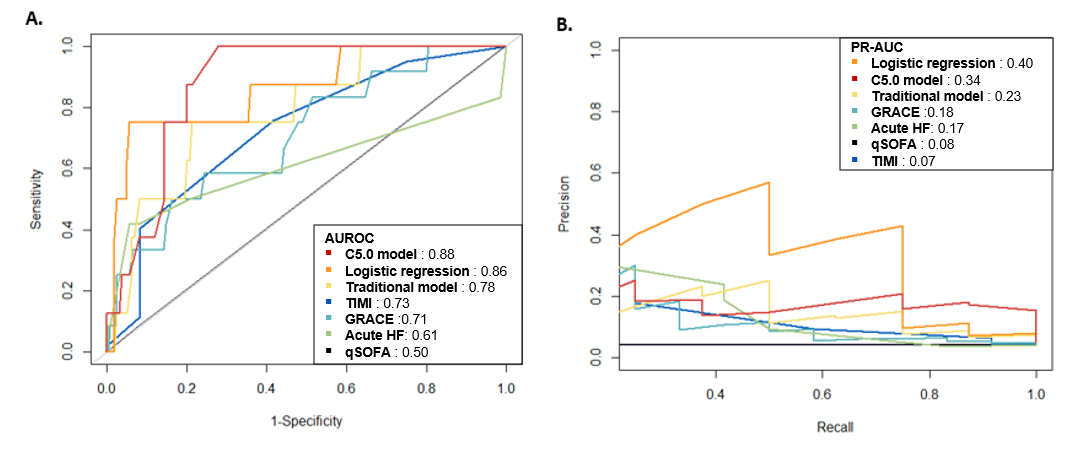
*

# *eTable 1*: Participating centres, number of patients enrolled and belonging cohort.

This table shows all participating centers with the respective number of patients recruited and the cohort (validation or index) in which the patients were analyzed.

| Centers | Patients recruited (N) | Corresponding cohort |
| --- | --- | --- |
| Amiens, University Hospital Center | 6 | Validation |
| Annecy, Hospital Center | 51 | Index |
| Avignon, Hospital Center | 58 | Index |
| Bobigny, Avicenne University Hospital Center, APHP | 9 | Index |
| Boulogne Billancourt, Hôpital Ambroise Pare, University Hospital Center, APHP | 10 | Index |
| Bordeaux, University Hospital Center | 45 | Index |
| Brest, University Hospital Center | 63 | Index |
| Caen, University Hospital Center | 32 | Validation |
| Chartres, Hospital Center | 30 | Index |
| Créteil, Henri Mondor University Hospital Center | 52 | Index |
| Dijon, University Hospital Center | 69 | Index |
| Fréjus, Hospital Center | 26 | Index |
| Grenoble, University Hospital Center | 76 | Index |
| La Réunion, University Hospital Center | 54 | Index |
| Lille, University Hospital Center | 43 | Validation |
| Limoges, University Hospital Center | 6 | Index |
| Lyon, University Hospital Center | 42 | Index |
| Marseille, La Timone University Hospital Center, APHM | 51 | Index |
| Martinique, Fort de France University Hospital Center | 33 | Index |
| Montfermeil, Hospital Center | 40 | Index |
| Montpellier, University Hospital Center | 46 | Validation |
| Montreuil, Hospital Center | 32 | Index |
| Neuilly sur Seine, Ambroise Paré Private Hospital | 42 | Validation |
| Nîmes, University Hospital Center | 56 | Validation |
| Orléans, Regional Hospital Center | 50 | Validation |
| Paris, Hôpital Bichat, University Hospital Center, APHP | 35 | Index |
| Paris, Hôpital Cochin, University Hospital Center, APHP | 30 | Index |
| Paris, Hôpital Européen Georges Pompidou, University Hospital Center, APHP | 12 | Index |
| Paris, Hôpital Lariboisiere, University Hospital Center, APHP | 60 | Index |
| Paris, Hôpital Saint-Antoine, University Hospital Center, APHP | 27 | Validation |
| Percy-Clamart, Hôpital d’Instruction des Armées | 10 | Index |
| Poitiers, University Hospital Center | 73 | Index |
| Rennes, University Hospital Center | 42 | Index |
| Rouen, University Hospital Center | 25 | Index |
| Saintes, Hospital Center | 25 | Index |
| Strasbourg, University Hospital Center | 84 | Index |
| Toulouse, University Hospital Center | 83 | Index |
| Tours, Clinique Saint Gatien Alliance (NCT+), Saint-Cyr-sur-Loire | 17 | Index |
| Versailles, Hôpital Mignot, Hospital Center | 30 | Index |
| Total | 1575 | . |

*Abbreviations : AP-HM: Assistance publique – Hôpitaux de Marseille; AP-HP: Assistance publique – Hôpitaux de Paris.*

| Type of variable | Detailed features |
| --- | --- |
| Demographics | Age, gender, BMI. |
| CV risk factors | Hypertension, dyslipidaemia, diabetes, known CAD, family history of CAD. |
| Medical history of non-CV disease | Active cancer, psychiatric history |
| Drug and substance use | Illicit drug use, alcohol consumption, CO level. |
| Clinical parameters at admission | Mean arterial pressure, heart rate, oxygen saturation, Killip class |
| Biological parameters at admission | Haemoglobin, creatinemia, troponin peak, NTproBNP |
| Echocardiographic data | LVEF, LA dilatation, RV dilatation, LV dilation, LV dysfunction, peak E/e’ ratio, TAPSE. |

*eTable 2.* Variables used in the feature selection algorithm.

*Abbreviations: BMI stands for Body Mass Index, CAD stands for Coronary Artery Disease, LA: Left Atrium, LV: Left Ventricular, LVEF: Left Ventricular Ejection Fraction.*

# *eTable 3.* Missing data in the final machine learning model

| Variables in the machine learning model | Missing data (%) |
| --- | --- |
| Ratio E/e’ | 26.3 |
| TAPSE | 13.8 |
| Exhaled CO | 7.5 |
| Left Ventricular Ejection Fraction | 2.5 |
| Mean arterial pressure | 0.4 |
| Killip class | 0.2 |
| Illicit drug use | 0.0 |

*eTable 4.* Compared selected features of the machine learning model in the training and internal validation sets within the index cohorts

| Variables (N, % unless otherwise specified) | Training  set  (N=844) | Internal validation set  (N=361) | P value |
| --- | --- | --- | --- |
| Drug and substance consumption |  |  |  |
| Illicit drug use | 86 (10.2) | 39 (10.8) | 0.83 |
| CO level, ppm (mean ± SD) | 4.74 ± 4.89  (N=781) | 5.30 ± 5.36  (N=361) | 0.11 |
| Clinical parameters at admission |  |  |  |
| Mean arterial pressure, mmHg (mean ± SD) | 98 ± 18  (N=840) | 99 ± 17  (N=361) | 0.42 |
| Killip Class | (N=842) | (N=360) | 0.34 |
| I | 701 (83.1) | 295 (81.7) |  |
| II | 88 (10.4) | 48 (13.3) |  |
| ≥ III | 53 (6.3) | 17 (4.7) |  |
| Echocardiographic data |  |  |  |
| LVEF, % (mean ± SD) | 52 ± 13  (N=823) | 52 ± 13  (N=355) | 0.64 |
| E/e' ratio, cm/s (mean ± SD) | 9.1 ± 4.1  (N=622) | 9.0 ± 4.1  (N=283) | 0.67 |
| TAPSE, mm (mean ± SD) | 20.8 ± 4.5  (N=727) | 20.9 ± 4.5  (N=318) | 0.76 |
| Outcomes |  |  |  |
| Major Adverse Events | 39 (4.6) | 20.9 (4.5) | 0.99 |

N values are detailed enter parenthesis when different from the total N.

Abbreviations: LVEF: Left Ventricular Ejection Fraction, SD: Standard Deviation.

# *eTable 5.* Compared characteristics of the external derivation cohort and index cohort.

| Variables (N, % unless otherwise specified) | External validation cohort  (N=294) | Index  cohort  (N=1205) | | P value | |  |
| --- | --- | --- | --- | --- | --- | --- |
| Demographic data |  |  | |  | |  |
| Age, years (mean ± SD) | 62.2±15.1 | 63.5±14.9 | | 0.18 | |  |
| Men | 196 (66.7) | 847 (70.3) | | 0.25 | |  |
| BMI, kg/m² (mean ± SD) | 27.4±5.5 | 27.2±5.52 | | 0.62 | |  |
| Cardiovascular risk factors |  |  | |  | |  |
| Diabetes | 64 (21.8) | 262 (21.7) | | 0.99 | |  |
| Hypertension | 147 (50.0) | 648 (53.8) | | 0.27 | |  |
| Dyslipidemia | 101 (34.4) | 481 (39.9) | | 0.09 | |  |
| Current smoker | 74 (40.7) | 306 (39.5) | | 0.84 | |  |
| Known CAD | 118 (40.1) | 405 (33.6) | | 0.042 | |  |
| Family history of CAD | 46 (15.6) | 201 (16.7) | | 0.73 | |  |
| Drug and substance consumption |  |  | |  | |  |
| Illicit drug use | 181 (63.3) | 618 (52.1) | | 0.41 | |  |
| Alcohol use at least once a week | 36 (12.2) | 125 (10.4) | | 0.001 | |  |
| CO level, ppm (mean ± SD) | 4.9±4.6 | 4.9±5.0 | | 0.94 | |  |
| Medical history of non-cardiovascular disease | |  | |  | |  |
| Active cancer | 8 (2.7) | 53 (4.4) | | 0.11 | |  |
| Psychiatric history | 36 (12.3) | 120 (10.0) | | 0.28 | |  |
| Clinical parameters at admission |  |  | |  | |  |
| Mean arterial pressure, mmHg (mean ± SD) | 98.3 (18.2) | 98.1 (17.6) | | 0.87 | |  |
| Heart rate, bpm (mean ± SD) | 84±24 | 82±24 | | 0.19 | |  |
| Oxygen saturation, % (mean ± SD) | 98±2 | 97±6 | | 0.009 | |  |
| Killip Class |  |  | | 0.13 | |  |
| I | 242 (82.3) | 996 (82.7) | |  | |  |
| II | 33 (11.2) | 136 (11.3) | |  | |  |
| ≥ III | 15 (5.1) | 70 (5.8) | |  | |  |
| Biological parameters at admission (mean ± SD) | | |  | |  | |
| Haemoglobin, g/dl | 13.8±1.8 | 13.5±2.0 | | 0.02 | |  |
| Creatinemia, µmol/l | 92.0±59.5 | 97.5±71.5 | | 0.17 | |  |
| High‐sensitivity cardiac troponin peak,Ul/l | 376±1186 | 1783±17549 | | 0.007 | |  |
| NTproBNP, pg/ml | 7237±10367 | 7948±16238 | | 0.35 | |  |
| Echocardiographic data |  |  | |  | |  |
| LVEF, %, (mean ± SD) | 51±13 | 52±13 | | 0.25 | |  |
| LV Dilatation | 24 (8.2) | 114 (9.5) | | 0.56 | |  |
| RV Dilatation | 24 (8.2) | 79 (6.6) | | 0.04 | |  |
| LA Dilatation | 43 (14.6) | 222 (18.4) | | 0.15 | |  |
| LV Dysfunction | 11 (3.7) | 91 (7.6) | | 0.03 | |  |
| E/A ratio, cm/s (mean ± SD) | 1.2±0.7 | 1.1±0.6 | | 0.12 | |  |
| E/e' ratio, cm/s (mean ± SD) | 8.8±3.8 | 9.1±4.1 | | 0.39 | |  |
| sPAP, mmHg (mean ± SD) | 34±14 | 35±14 | | 0.21 | |  |
| TAPSE, mm (mean ± SD) | 20.8±4.7 | 20.9±4.5 | | 0.94 | |  |
| LVOT VTI, cm (mean ± SD) | 19±6 | 19±5 | | 0.10 | |  |
| Outcomes |  |  | |  | |  |
| Major adverse events | 12 (4.1) | 55 (4.6) | | 0.84 | |  |

Abbreviations: BMI: Body Mass Index, CAD: Coronary Artery Disease, LA: Left Atrium, LV: Left Ventricle, LVEF: Left Ventricular Ejection Fraction, LVOT VTI: Left Ventricular Outflow Tract Velocity Time Integral, RV: Right Ventricle, SD: Standard Deviation, sPAP: systolic Pulmonary Artery Pressure.
